# Therapeutic efficacy and effects of artesunate-amodiaquine and artemether-lumefantrine on malaria-associated anaemia in Nigerian children aged two years and under

**DOI:** 10.1186/s40249-016-0165-2

**Published:** 2016-07-06

**Authors:** Akintunde Sowunmi, Kazeem Akano, Adejumoke I. Ayede, Godwin Ntadom, Elsie O. Adewoye, Bayo Fatunmbi, Temitope Aderoyeje

**Affiliations:** Department of Pharmacology and Therapeutics, University of Ibadan, Ibadan, Nigeria; Institute for Medical Research and Training, University of Ibadan, Ibadan, Nigeria; Department of Paediatrics, University of Ibadan, Ibadan, Nigeria; Malaria Elimination Programme, Federal Ministry of Health, Abuja, Nigeria; Department of Physiology, University of Ibadan, Ibadan, Nigeria; World Health Organization, Regional Office for the Western Pacific, Phnom Penh, Cambodia; Department of Clinical Pharmacology, University College Hospital, Ibadan, Nigeria

**Keywords:** Falciparum malaria, Malaria-related anaemia, Young children, Artemisinin-based combination therapies, Nigeria

## Abstract

**Background:**

Artemisinin-based combination therapies are recommended as first-line treatments for uncomplicated falciparum malaria, but there is little evaluation of their efficacy and effects on uncomplicated malaria-associated anaemia in children aged 2 years and under.

**Methods:**

Parasitological efficacy and effects on malaria-associated anaemia were evaluated in 250 malarious children aged 2 years and under, and efficacy was evaluated in 603 malarious children older than two but younger than 5 years of age following treatment with artesunate-amodiaquine (AA) or artemether-lumefantrine (AL). Kinetics of the disposition of parasitaemia following treatment were evaluated using a non-compartment model. Late-appearing anaemia (LAA) was diagnosed using the following criteria: clearance of parasitaemia, fever and other symptoms occurring within 7 days of starting treatment, adequate clinical and parasitological response on days 28–42, haematocrit (HCT) ≥ 30 % at 1 and/or 2 weeks, a fall in HCT to < 30 % occurring at 3–6 weeks, absence of concomitant illness at 1–6 weeks, and absence of asexual parasitaemia detected using both microscopy and polymerase chain reaction (PCR) at 1–6 weeks.

**Results:**

Overall, in children aged 2 years and under, the PCR-corrected parasitological efficacy was 97.2 % (95 % *CI* 92.8–101.6), which was similar for both treatments. In children older than 2 years, parasitological efficacy was also similar for both treatments, but parasite prevalence 1 day after treatment began was significantly higher, and fever and parasite clearance times were significantly faster in the AA-treated children compared with the AL-treated children. Declines in parasitaemia were monoexponential with an estimated elimination half-time of 1 h. Elimination half-times were similar for both treatments. In children aged 2 years and under who were anaemic at presentation, the mean anaemia recovery time was 12.1 days (95 % *CI* 10.6–13.6, *n* = 127), which was similar for both treatments. Relatively asymptomatic LAA occurred in 11 children (4.4 %) aged 2 years and under, the recovery from which was uneventful.

**Conclusion:**

This study showed that AA and AL are efficacious treatments for uncomplicated falciparum malaria in Nigerian children aged 2 years and under, and that AA clears parasitaemia and fever significantly faster than AL in children older than 2 years. Both treatments may cause a relatively asymptomatic LAA with uneventful recovery in a small proportion of children aged 2 years and under.

**Trials registration:**

Pan African Clinical Trial Registry PACTR201508001188143, 3 July 2015; PACTR201510001189370, 3 July 2015; PACTR201508001191898, 7 July 2015 and PACTR201508001193368, 8 July 2015 http://www.pactr.org.

**Electronic supplementary material:**

The online version of this article (doi:10.1186/s40249-016-0165-2) contains supplementary material, which is available to authorized users.

## Multilingual abstracts

Please see Additional file 1 for translations of the abstract into the five official working languages of the United Nations.

## Background

Artemisinin-based combination therapies (ACTs) have remained efficacious since their adoption as first-line treatments for uncomplicated *Plasmodium falciparum* malaria [1]. In Nigeria, artemether-lumefantrine (AL) and artesunate-amodiaquine (AA) were adopted as first-line treatments in 2005 [2]. However, recent reports of artemisinin resistance in *P. falciparum* malaria [3] in the Greater Mekong subregion [4–9], and its declining responsiveness reported in Kenya [10] and Suriname [11], have made it imperative to monitor, on a continuous basis, the efficacy of ACTs for treating *P. falciparum* malaria globally.

Traditionally, in endemic areas, antimalarial efficacy assessments have been carried out in children younger than 5 years of age because of this age group’s relative lack of antimalarial immunity [12]. However, it is unclear if very young children (≤2 years old) differ in therapeutic responses from those older than two but younger than 5 years of age. Additionally, there has been little or no evaluation of the efficacy of these drug combinations in children with uncomplicated falciparum malaria who are aged 2 years and under. Such information is necessary as it may aid in the early detection of declining responsiveness of *P. falciparum* malaria infections to ACTs in children, as well as help guide appropriate responses.

In the present study, we analyzed the responsiveness of *P. falciparum* infections to AA and AL in the 10-year period since the adoption of these as first-line treatments for uncomplicated falciparum malaria in Nigeria. We compared the therapeutic responses to the two ACTs in children aged 2 years and under with children older than 2 years of age. In addition, we evaluated the recovery from malaria-associated anaemia and described late-appearing anaemia (LAA) in children aged 2 years and under.

## Methods

### Study sites

**Fig. 1:**
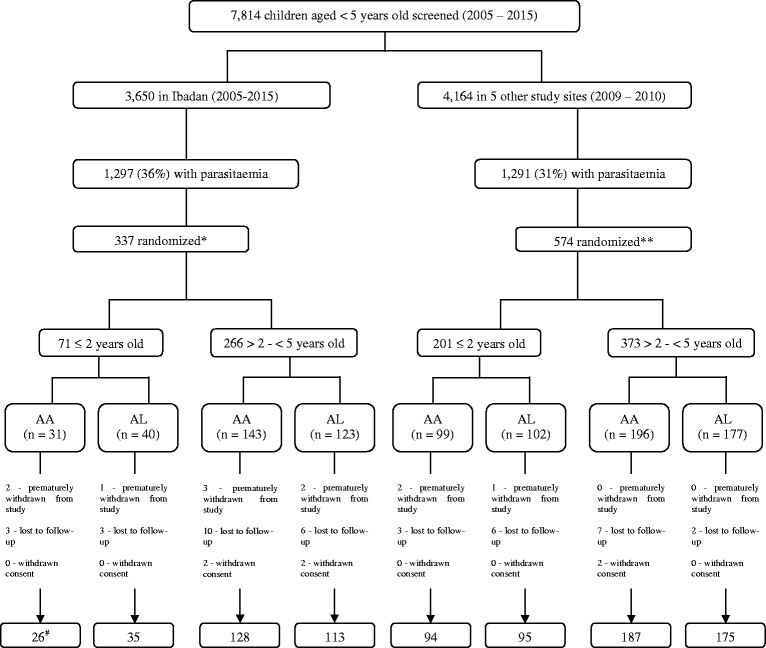
Study profile. AA, artesunate-amodiaquine; AL, artemether-lumefantrine, *follow-up was for 42 days, **follow-up was for 28 days, #number of children that completed follow-up period

The study was conducted between October 2009 and November 2010 at the following sites in Nigeria: Agbani in Enugu State, Barkin Ladi in Plateau State, and Damboa in Borno State (the eastern flank of the study sites); Ijede in Lagos State and Makarfi in Kaduna State (the western flank of the study sites); and in Sabo quarters of Ibadan, Oyo State, located on the western flank between 2005 and 2015 (see Fig. 1).

In virtually all study locations, malaria is hyperendemic and transmission occurs all year round, however, it is more intense during the rainy season, which lasts from April to October. *P. falciparum* is the predominant species, accounting for over 98 % of all infections [1, 13]. Children are more affected than adults and asymptomatic infections more often occur in older school children aged greater than 5 years and adults [13]. The details pertaining to the larger studies conducted have been reported elsewhere [14–20].

#### Study procedure

Specific study procedures have been described for each study conducted before [14–20]. All enrolment procedures are the same at all sites. Patients were eligible for evaluation if they were: aged 6–24 months or 25–59 months, had symptoms compatible with acute uncomplicated *P. falciparum* malaria with a monoinfection ≥ 1 000 μL^−1^ of blood, no history of antimalarial drug ingestion in the 2 weeks prior to enrolment, absence of severe malaria and obtained written informed consent from parents or guardians.

Enrolled patients were randomized and received AL or AA (co-formulated). Artemether-lumefantrine (Coartem®, Novartis, Basel, Switzerland) was administered according to body weight: patients weighing 5–14 kg received one tablet; those weighing 15–24 kg received two tablets at presentation (0 h), 8 h later, and at 24, 36, 48 and 60 h after the first dose. Each tablet of AL contained 20 mg of artemether and 120 mg of lumefantrine. Artesunate-amodiaquine (Coarsucam®, Sanofi Aventis, France) was also administered according to body weight: patients weighing from 4.5 to under 9 kg received one tablet of 25 mg/67.5 mg of a fixed-dose combination of artesunate/amodiaquine respectively, those weighing 9 to under 18 kg received one tablet of 50 mg/135 mg, and those weighing 18 to under 24 kg received one tablet of 100 mg/270 mg, daily for 3 days. Children older than 2 years were treated with AA or AL, as previously described [20].

All drugs were given orally. All doses of AA were given by direct observed therapy. The doses of AL at presentation (day 0, 0 h), and at 8, 24 and 48 h were administered by direct observed therapy. Doses of AL at 36 and 60 h were given by parents/guardians at home.

Thick and thin blood films were obtained from each child as soon as he/she came to the clinic, and the slides were carefully labeled with the patients’ codes and air-dried before being stained. The slides were examined by light microscopy under an oil-immersion objective lens at 1000× magnification by two independent assessors who did not know the drug regimen of the patients. A senior member of the study team reviewed the slides if there was any disagreement between the two microscopists. In addition, the slides of every fourth child were reviewed by the senior member. Parasitaemia, asexual or sexual, was estimated by counting the asexual and sexual parasites relative to 500 leukocytes, or 500 asexual or sexual forms, whichever occurred first, in the thick films. Using this figure, the parasite density was calculated assuming a leukocyte count of 6 000 μL^−1^ of blood. A slide was considered parasite negative if no asexual or sexual parasite was detected after examination of 200 microscopic fields. A clinical and parasitological evaluation was done on days 1–3 and on days 7, 14, 21 and 28 at all study sites, except in Ibadan where additional follow-up was done on days 35 and 42.

Cure rates on day 28 were adjusted based on the polymerase chain reaction (PCR) genotyping results of patients’ recurrent parasitaemia paired samples 7 days after starting treatment, as previously described [20]. An asexual parasite reduction ratio (PRR) [21] was defined as the ratio of day 0/day 2 parasitaemia (and for convenience, referred to as PRR_D2_): that is, $PR{R}_{D2}=\left.\frac{Parasitaemia\  on\  day\ 0}{Parasitaemia\  on\  day\ 2}\right]$. The asexual PRR on day 1 (PRR_D1_) was defined as the ratio of day 0/day 1 parasitaemia: that is, $PR{R}_{D1}=\left.\frac{Parasitaemia\  on\  day\ 0}{Parasitaemia\  on\  day\ 1}\right]$.

#### Kinetics of the disposition of parasitaemia following treatment

In 20 children aged 2 years and under, enrolled between 2008 and 2015 at the Ibadan study site, clinical and parasitological evaluations were done at the following times: at 0, 1, 2, 4, 6, 8 and 24 h, and on days 2–7, 14, 21, 28, 35 and 42. The kinetics of the time-course of the parasitaemia was estimated using a non-compartmental model, as previously described [17, 22].

#### Haematological evaluation

Capillary blood collected before and during follow-up was used to measure haematocrit (HCT) using a microhaematocrit tube and microcentrifuge (Hawksley, Lancing, UK). Anaemia was defined as HCT < 30 %, and was classified as either mild, moderate or severe if HCT was 21–29, 15–20 or < 15 %, respectively. Anaemia recovery time (AnRT) (in anaemic patients) was defined as the time elapsed from drug administration to getting a HCT value ≥ 30 % [18, 20, 23, 24].

#### Diagnosis of LAA after administering ACTs

In children aged 2 years and under, a diagnosis of LAA was made if the following criteria were met following the initiation of ACTs: clearance of parasitaemia, fever and other symptoms occurring within seven days of starting treatment, adequate clinical and parasitological response (ACPR) [25, 26] on days 28–42, HCT ≥ 30 % at 1 and/or 2 weeks, a fall in HCT to < 30 % occurring at 3–6 weeks, absence of concomitant illness at 1–6 weeks, and absence of asexual parasitaemia detected using both microscopy and PCR at 1–6 weeks (Sowunmi et al., 2015, unpublished, submitted for publication).

#### Statistical analysis

Data were analyzed using the Epi Info™ version 6 software (Centers for Disease Control and Prevention, Atlanta, GA, USA) [27] and the statistical program SPSS for Windows version 20.0 (SPSS Inc., Chicago IL, USA) [28]. Variables considered in the analysis were related to the densities of *P. falciparum* asexual and sexual forms. Proportions were compared by calculating *χ*^2^ using Yates’s correction, Fisher’s exact or Cochran-Mantel-Haenszel tests. Normally distributed, continuous data were compared using the Student’s *t*-test and analysis of variance (ANOVA). Data not conforming to a normal distribution were compared using the Mann-Whitney *U* test and the Kruskal-Wallis H test (or by Wilcoxon rank sum test). The relationship between two continuous variables was assessed using the Spearman’s rank correlation coefficient. The cumulative risk of parasite reappearance was calculated by survival using the Kaplan-Meier method. *P*-values of < 0.05 were considered statistically significant. Data were double-entered serially using patients’ codes and were only analyzed at the end of the study.

#### Ethical clearance

The study protocol was approved by the Ministry of Health, Ibadan and the National Health Research Ethics Committee, Abuja, Nigeria. Written informed consents were obtained from parents/guardians of the children.

## Results

### Patient characteristics at enrolment

During the study period, a total of 911 children aged younger than 5 years from five geographical areas of Nigeria who had parasitaemia were enrolled and treated with AA or AL. Of these, 272 (30 %) were aged 2 years and under (see Fig. 1) and of those, 250 were evaluated in this study. The characteristics of the children at enrolment in each study site and for each year of study are shown in Table 1.

**Table 1:** Baseline characteristics of ≤ 2-year old malarious children at enrolment according to year and site of study

Study site (year of study)^a^	No. of children	Gender	Age (year)		Weight (kg)	Duration (days)	Temperature (°C)			Haematocrit (%)		Degree of anaemia			Parasitaemia (μL^−1^)	
		M/F	Mean ± sd (Range)	No. <1 year	Mean ± sd (Range)	Mean ± sd (Range)	Mean ± sd (Range)	No. >37.4 °C	No. ≥40 °C	Mean ± sd (Range)	No. < 30 %	Mild	Moderate	Severe	Geometric mean (Range)	No. >100,000 μL^−1^
Ibadan (2005–2010)	47	26/21	1.7 ± 0.4 (0.7–2)	4	9.7 ± 2.5 (6–17)	2.9 ± 1.5 (1–7)	38.5 ± 1.1 (36.1–40.8)	39	2	28.3 ± 4.5 (20–38)	26	23	3	-	30,924 (1,614–304,500)	7
Damboa (2009–2010)	24	16/8	1.5 ± 0.5 (0.5–2)	7	9.9 ± 3.9 (5–20)	-	37.9 ± 0.6 (37.5–39.3)	24	-	27.9 ± 5.9 (14–36)	12	9	2	1	5,988 (1,000–123,774)	1
Agbani (2009–2010)	57	24/33	1.3 ± 0.5 (0.5–2)	20	9.9 ± 1.7 (5–17)	-	37.6 ± 1.1 (35.6–40.3)	30	1	27.3 ± 4.3 (18–40)	38	33	5	-	10,343 (1,018–155,520)	3
Makarfi (2009–2010)	41	24/17	1.4 ± 0.4 (0.5–2)	15	8.4 ± 1.6 (5–12)	-	37.9 ± 1 (36–39.4)	29	-	29.4 ± 5.1 (16–37)	15	13	2	-	10,208 (1,301–112,421)	2
Ijede (2009–2010)	28	18/10	1.4 ± 0.4 (0.6–2)	6	9.2 ± 1.6 (5–12)	-	38.1 ± 1.4 (36–40.3)	18	4	28.9 ± 4.8 (19–38)	16	14	2	-	10,788 (1,071–78,769)	-
Barkin Ladi (2009–2010)	39	20/19	1.6 ± 0.5 (0.6–2)	8	10.6 ± 2.3 (7–15)	3.1 ± 1 (1–7)	37.9 ± 1 (36.1–40)	30	1	30.7 ± 4.2 (22–40)	13	13	-	-	17,336 (2,132–104,154)	1
Ibadan (2011–2015)	14	11/3	1.4 ± 0.6 (0.5–2)	5	9.2 ± 2.8 (5–14)	3 ± 0.7 (2–4)	38.1 ± 1.3 (36.3–40)	9	1	29 ± 4.1 (22–37)	7	7	-	-	23,326 (2,924–114,504)	-
ALL (2005–2015)	250	139/111	1.5 ± 0.5 (0.5–2)	65	9.6 ± 2.3 (5–20)	3 ± 1.2 (1–7)	38 ± 1.1 (35.6–40.8)	179	9	28.7 ± 4.8 (14–40)	127	112	14	1	13,714 (1000–304,500)	16
*P* value	-	0.14	0.03	0.03	0.001	0.83	0.01	0.02	0.29	0.03	0.02	0.85	0.99	-	<0.0001	0.11

*sd* standard deviation

^a^artesunate-amodiaquine and artemether-lumefantrine were evaluated at all sites

Children enrolled in Ibadan between 2005 and 2010 but not between 2011 and 2015, were significantly older and had a significantly higher body temperature and enrolment parasitaemia compared with children enrolled at the other study sites. Children enrolled in Barkin Ladi had a significantly higher HCT at enrolment. In children aged 2 years and under, anaemia at presentation was more significantly common in children older than 1 year of age compared with children aged 1 year and under (103/185 versus 25/65; *P* <0.05).

### Therapeutic responses

#### Children ≤ 2 years old

In children aged 2 years and under, the overall PCR-corrected ACPR on day 28 was 97.2 % (95 % *CI* 92.8–101.6). Overall, for both treatments and for all study sites, the PCR-uncorrected ACPR on day 28 was 234/250 (94 %) and the PCR-corrected ACPR on day 28 was 234/247 (95 %) (see Table 2).

**Table 2:** Overall efficacy of artesunate-amodiaquine or artemether-lumefantrine in ≥2 years old malarious children according to study sites

	PCR uncorrected			PCR corrected			
Site (Year)	ACPR_u	Failure_u	Total	ACPR_c	Recrudescence	Total	% Recrudescence
Ibadan (2005–2010)	47	0	47	47	0	47	-
Damboa (2009–2010)	24	0	24	24	0	24	-
Agbani (2009–2010)	50	7	57	50	6	56	10.7
Makarfi (2009–2010)	40	1	41	40	0	40	-
Ijede (2009–2010)	27	1	28	27	0	27	-
Barkin Ladi (2009–2010)	32	7	39	32	3	35	8.6
Ibadan (2011–2015)	14	0	14	14	0	14	-
Total (2005–2015)	234	16	250	234	9	247	3.6

*PCR* polymerase chain reaction, *ACPR_u* adequate clinical and parasitological response uncorrected, *ACPR_c* adequate clinical and parasitological response corrected, *Failure_u* treatment failure uncorrected

Table 3 shows the therapeutic responses to AA and AL for each study site. For AA, the overall PCR-corrected ACPR on day 28 was 99.3 % (95 % *CI* 97.6–101) at all sites. The PCR-uncorrected ACPR on day 28 was 112/120 (93 %) and the PCR-corrected ACPR on day 28 was 112/116 (97 %). The PCR-corrected ACPR was similar at all sites. For AL, the overall PCR-corrected ACPR on day 28 was 96.9 % (95 % *CI* 91.5–102.3) at all sites. The PCR-uncorrected ACPR on day 28 was 122/130 (94 %) and the PCR-corrected ACPR on day 28 was 122/127 (96 %). The PCR-corrected ACPR was similar at all sites. Overall, the PCR-corrected ACPR on day 28 was similar for both treatments (99.3 % versus 96.9 % for AA and AL, respectively; *P* >0.05).

**Table 3:** Efficacy of artesunate-amodiaquine or artemether-lumefantrine in ≥2 years old malarious children according to study site and year of enrolment

Site (Year)	PCR uncorrected			PCR corrected			
	ACPR_u	Failure_u	Total	ACPR_c	Recrudescence	Total	% Recrudescence
Artesunate-amodiaquine							
Ibadan (2005–2010)	15	0	15	15	0	15	-
Damboa (2009–2010)	17	0	17	17	0	17	-
Agbani (2009–2010)	23	5	28	23	4	27	14.8
Makarfi (2009–2010)	17	0	17	17	0	17	-
Ijede (2009–2010)	14	0	14	14	0	14	-
Barkin Ladi (2009–2010)	15	3	18	15	0	15	-
Ibadan (2011–2015)	11	0	11	11	0	11	-
Total (2005–2015)	112	8	120	112	4	116	3.4
Artemether-lumefantrine							
Ibadan (2005–2010)	32	0	32	32	0	32	-
Damboa (2009–2010)	7	0	7	7	0	7	-
Agbani (2009–2010)	27	2	29	27	2	29	6.9
Makarfi (2009–2010)	23	1	24	23	0	23	-
Ijede (2009–2010)	13	1	14	13	0	13	-
Barkin Ladi (2009–2010)	17	4	21	17	3	20	15
Ibadan (2011–2015)	3	0	3	3	0	3	-
Total (2005–2015)	122	8	130	122	5	127	3.9

*PCR* polymerase chain reaction, *ACPR_u* adequate clinical and parasitological response uncorrected, *ACPR_c* adequate clinical and parasitological response corrected, *Failure_u* treatment failure uncorrected

No child had early treatment failure, however, late parasitological failure (LPF) occurred in 16 children, who were all enrolled between 2005 and 2010. There was no significant difference in the proportions of children with LPF in the two treatment groups (8/120 children treated with AA versus 8/130 children treated with AL, *P* >0.05). The proportion of children with LPF was also similar in infants and non-infants, and was not related to their anaemia status at presentation (4/65 versus 12/185 in infants and non-infants, respectively, *P* >0.05; and 10/127 versus 6/123 in anaemic and non-anaemic children, respectively, *P* >0.05).

**Fig. 2:**
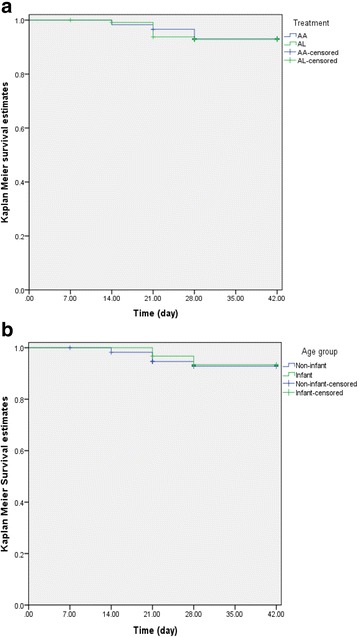
Kaplan-Meier survival estimates of asexual parasitaemia (**a**) after treatment of ≤ 2 years old children with AA (*blue line*) or AL (*green line*; log-rank statistic = 0.006; *P* = 0.94) and (**b**) in infants (*green line*) and non-infants (*blue line*) treated with AA or AL (log-rank statistic = 0.03; *P* = 0.86). AA, artesunate-amodiaquine; AL, artemether-lumefantrine

Recrudescence occurred in nine children (six from Agbani and three from Barkin Ladi). Recrudescence occurred in four children treated with AA and five children treated with AL. Overall, mean time to recrudescence was 21.8 ± 5.5 days (range 14–28) and was similar in children treated with AA and AL (22.8 ± 6.7 days [range 14–28] versus 21 ± 4.9 days [range 14–28]; *P* = 0.66). The probabilities of the reappearance of asexual parasitaemia after treatment were similar between AA and AL (log-rank statistic = 0.006, *P* = 0.94, see Fig. 2a). The probabilities of reappearance of asexual parasitaemia after treatment with both drugs were similar in infants and non-infants (log-rank statistic = 0.03, *P* = 0.86, see Fig. 2b).

Table 4 shows that other measures of therapeutic responses were similar in children treated with AA and AL. Only three children (two on AA and one on AL) exhibited parasitaemia on day 3.

**Table 4:** Other measures of treatment outcomes in children ≤ 2 treated with artesunate-amodiaquine or artemether-lumefantrine

	ALL (250)	Artesunate-amodiaquine (120)	Artemether-lumefantrine (130)	*P* value
Parasite prevalence on day 1	126	58	68	0.62
Parasite positivity rate on day 3	3	2	1	0.61
PRR_D1_				
Geometric mean	5.5 × 10^2^	5.6 × 10^2^	5.4 × 10^2^	0.79
Range	1.1 × 10^−1^–2.9 × 10^5^	1.1 × 10^−1^–1.3 × 10^5^	4.1 × 10^−1^–2.9 × 10^5^	
PRR_D2_				
Geometric mean	1.1 × 10^4^	1.0 × 10^4^	1.1 × 10^4^	0.7
Range	1.9 × 10^1^–3.0 × 10^5^	1.9 × 10^1^–1.3 × 10^5^	2.6 × 10^1^–3.0 × 10^5^	
Fever clearance time (day)				
Mean	1.3	1.3	1.4	0.83
95 % CI	1.2–1.5	1.1–1.5	1.1–1.6	
Parasite clearance time (day)				
Mean	1.6	1.5	1.6	0.46
95 % CI	1.5–1.7	1.4–1.7	1.5–1.7	

*PRR*_*D1*_ parasite reduction ratio 1 day after treatment started, *PRR*_*D2*_ parasite reduction ratio 2 days after treatment started, *ALL* all children

#### Children ≤ 2 years old at the Ibadan study site

Overall, the therapeutic responses of 61 children aged 2 years and under were evaluated during the 10-year period since AA and AL were introduced as first-line treatments for malaria in Nigeria (47 children from 2005 to 2010 and 14 children from 2011 to 2015). Of the 61 children, 26 were treated with AA and 35 with AL. Because of the small number of patients, therapeutic responses over the years were merged for both treatments. Baseline parameters at enrolment were similar for both AA and AL. Mean age (1.6 ± 0.5 years versus 1.6 ± 0.4 years, *P* = 0.43) and geometric mean parasitaemia (28,143 μL^−1^ [1614–128,976] versus 29,629 μL^−1^ [4938–304,500]; *P* = 0.68) were similar for the AA and AL treatment groups, respectively. Therapeutic responses were also similar for the two treatments: ACPR on day 28 (26/26 versus 35/35), geometric mean PRR (1.9 × 10^4^ [range 2.2 × 10^2^–1.3 × 10^5^] versus 4.9 × 10^3^ [range 1.7 × 10^0^–2.9 × 10^5^]; and 2.8 × 10^4^ [range 1.6 × 10^3^– 1.3 × 10^5^] versus 2.8 × 10^4^ [range 1.9 × 10^3^–3.0 × 10^5^] one and 2 days after treatment began, respectively) and fever clearance time (FCT) (1.2 ± 0.1 standard error of mean, SEM [range 1–4, *n* = 22] versus 1.5 ± 0.2 SEM [1–7, *n* = 26]). The parasite clearance time (PCT) was significantly shorter for children treated with AA than for those treated with AL (1.1 ± 0.3 [range 1–2] versus 1.3 ± 0.5 [range 1–3]).

During the two study periods (2005–2010 and 2011–2015), the proportions of children with ACPR on day 28 (47/47 versus 14/14), parasite prevalence 1 day after treatment began (8/47 versus 3/14), PRRs (9.0 × 10^3^ [range 1.7 × 10^0^–2.9 × 10^5^] versus 7.4 × 10^3^ [range 1.2 × 10^2^–1.1 × 10^5^], and 3.1 × 10^4^ [range 1.6 × 10^3^–3.0 × 10^5^] versus 2.0 × 10^4^ [range 1.9 × 10^3^–1.1 × 10^5^] 1 and 2 days after treatment began, respectively), FCTs (1.4 ± 0.2 SEM [range 1–7, *n* = 39] versus 1.3 ± 0.3 SEM [1–4, *n* = 9]), and PCTs (1.2 ± 0.4 [range 1–2] versus 1.3 ± 0.6 [range 1–3]) were similar.

### Therapeutic responses according to treatment and age groups

#### Comparison of therapeutic responses in children ≤ 2 and > 2– < 5 years

Comparing all children treated with AA or AL showed that the PRRs one and 2 days after treatment began were significantly higher in children older than 2 years but younger than 5 years of age than in children aged 2 years and under (1.1 × 10^3^ [range 3.2 × 10^−1^–2.1 × 10^6^] versus 5.5 × 10^2^ [range 1.1 × 10^−1^–2.9 × 10^5^], *P* = 0.01; and 1.9 × 10^4^ [range 1.7 × 10^1^–2.1 × 10^6^] versus 1.1 × 10^4^ [range 1.9 × 10^1^–3.0 × 10^5^]; *P* < 0.0001; respectively). Fever clearance time and AnRT were significantly lower in children aged older than 2 years but younger than 5 years compared with children aged 2 years and under (1.1 days [95 % *CI* 1.1–1.2, *n* = 179] versus 1.3 days [95 % *CI* 1.2–1.4, *n* = 450], *P* = 0.02; and 10.7 days [95 % *CI* 9.6–11.7, *n* = 179] versus 12.8 days [95 % *CI* 11.1–14.4, *n* = 450], *P* <0.03; respectively). Other measures of therapeutic responses, for example, the PCR-corrected ACRP on day 28 (234/244 [96.3 %] versus 559/589 [94.9 %], *P* > 0.05); parasite prevalence 1 day after treatment began (126/250 [50 %] versus 278/603 [46 %], *P* > 0.05); and parasite positivity rate on day 3 (3/250 versus 7/603, *P* > 0.05), respectively, were similar in children aged 2 years and under and those older than 2 years but younger than 5 years.

**AA:** In children younger than 1 year old (*n* = 29) and those 1 year old and above (*n* = 91) who were treated with AA, no significant differences were observed in the therapeutic responses to the drug. In children aged 2 years and under (*n* = 120) and those older than 2 years but younger than 5 years (*n* = 315) who were treated with AA, PRRs 1 and 2 days after treatment began were significantly higher. The FCT was significantly lower in children older than 2 years but younger than 5 years of age compared with children aged 2 years and under (see Table 5).

**Table 5:** Therapeutic outcomes based on age group in children treated with artesunate-amodiaquine

Parameter	≤2 years			<5 years		
	<1 year (*n* = 29)	≥1 year (*n* = 91)	P value	≤2 years (*n* = 120)	≥2– < 5 years (*n* = 315)	*P* value
Haematocrit (%)						
Mean	28.8	28.4	0.63	28.5	30.5	<0.0001
95 % CI	27.4–30.3	27.2–29.5		27.6–29.4	30–31.1	
No <30 %	13	55	0.21	68	117	<0.0001
Parasitaemia (μL^−1^)						
Geometric mean	13,807	11,883	0.55	12,322	23,733	<0.0001
Range	1,018–114,504	1,000–128,976		1,000–128,976	1,000–1,125,000	
No. >100,000 μL^−1^	2	7	1.0	9	55	0.01
No. >250,000 μL^−1^	0	0	-	0	12	-
ACPR on day 28 (%)	26/27 (96)	86/89 (97)	1.0	112/116 (97)	294/310 (95)	0.48
Parasite prevalence on day 1 (%)	18 (62)	40 (44)	0.14	58 (48)	124 (39)	0.11
PRR_D1_						
Geometric mean	3.8 × 10^2^	6.4 × 10^2^	0.41	5.4 × 10^2^	1.9 × 10^3^	0.001
Range	3.9 × 10^0^–1.1 × 10^5^	1.1 × 10^−1^–1.3 × 10^5^		1.1 × 10^−1^–1.3 × 10^5^	3.2 × 10^−1^–1.1 × 10^6^	
PRR_D2_						
Geometric mean	1.0 × 10^4^	1.0 × 10^4^	0.56	1.0 × 10^4^	1.9 × 10^4^	0.001
Range	3.9 × 10^1^–1.1 × 10^5^	9.5 × 10^1^–1.3 × 10^5^		1.9 × 10^1^–1.3 × 10^5^	5.3 × 10^1^–1.1 × 10^6^	
Parasite positivity rate on day 3	0	3	-	2	2	0.31
Fever clearance time (day)						
Mean	1.3	1.3	0.79	1.3	1.1	0.005
95 % CI	1–1.6	1–1.7		1.1–1.5	1–1.1	
Parasite clearance time (day)						
Mean	1.7	1.5	0.06	1.5	1.4	0.11
95 % CI	1.5–1.9	1.4–1.6		1.4–1.6	1.4–1.5	
Anaemia recovery time (day)						
Mean	12.1	12.5	0.91	12.4	10.4	0.12
95 % CI	6–18.2	9.9–15.1		10.1–14.7	9–11.8	

*ACPR* adequate clinical and parasitological response, *PRR*_*D1*_ parasite reduction ratio 1 day after treatment started, *PRR*_*D2*_ parasite reduction ratio 2 days after treatment started

**AL:** In children younger than 1 year (*n* = 46) and those 1 year or above (*n* = 84) who were treated with AL, the parasite prevalence 1 day after treatment began and PCTs were significantly lower in children younger than 1 year compared with children 1 year or older. In children aged 2 years and under (*n* = 130) and those older than 2 years but younger than 5 years (*n* = 288) who were treated with AL, the PRR 2 days after treatment began was significantly lower in children older than 2 years but younger than 5 years compared with children aged 2 years and under (see Table 6).

**Table 6:** Therapeutic outcomes based on age group in children treated with artemether-lumefantrine

Parameter	≤2 years (*n* = 130)			<5 years (*n* = 418)		
	<1 year (*n* = 46)	≥1 year (*n* = 84)	*P* value	≤2 years (*n* = 130)	≥2– < 5 years (*n* = 288)	*P* value
Haematocrit (%)						
Mean	28.9	28.9	0.96	28.9	29.9	0.05
95 % CI	27.4–30.3	28–29.8		28.1–29.7	29.3–30.5	
No <30 %	19	40	0.61	59	129	0.99
Parasitaemia (μL^−1^)						
Geometric mean	11,981	17,207	0.16	15,138	23,001	0.003
Range	1,000–304,500	1108–288,461		1,000–305,500	1,030–2,124,000	
No. >100,000 μL^−1^	1	6	0.43	7	50	0.002
No. >250,000 μL^−1^	1	1	1.0	2	8	0.73
ACPR on day 28 (%)	45/46 (98)	77/81 (95)	0.65	122/127 (96)	265/279 (95)	0.82
Parasite prevalence on day 1	18 (39)	60	0.04	68 (52)	154 (53)	0.91
PRR_D1_						
Geometric mean	9.1 × 10^2^	4.0 × 10^2^	0.31	5.4 × 10^2^	5.6 × 10^2^	1.0
Range	1.7 × 10^0^–8.7 × 10^4^	4.1 × 10^−1^–2.9 × 10^5^		4.1 × 10^−1^–2.9 × 10^5^	9.5 × 10^−1^–2.1 × 10^6^	
PRR_D2_						
Geometric mean	1.1 × 10^4^	1.2 × 10^4^	0.51	1.1 × 10^4^	1.8 × 10^4^	0.004
Range	3.2 × 10^1^–3.0 × 10^5^	2.6 × 10^1^–2.9 × 10^5^		2.6 × 10^1^–3.0 × 10^5^	1.7 × 10^1^–2.1 × 10^6^	
Parasite positivity rate on day 3	0	1	-	1	1	0.53
Fever clearance time (day)						
Mean	1.2	1.3	0.64	1.3	1.2	0.4
95 % CI	1.1–1.4	1–1.6		1.1–1.5	1.1–1.3	
Parasite clearance time (day)						
Mean	1.4	1.7	0.01	1.6	1.6	0.92
95 % CI	1.3–1.6	1.6–1.8		1.5–1.7	1.5–1.7	
Anaemia recovery time (day)						
Mean	15.3	12.5	0.29	13.2	11	0.12
95 % CI	10.6–20.1	9.7–15.3		10.9–15.6	9.4–12.6	

*ACPR* adequate clinical and parasitological response, *PRR*_*D1*_ parasite reduction ratio 1 day after treatment started, *PRR*_*D2*_ parasite reduction ratio 2 days after treatment started

#### Comparison of therapeutic responses in children aged > 2– < 5 years

Three hundred and fifteen children and 288 children older than 2 years of age were treated with AA and AL, respectively. The parasite prevalence 1 day after treatment began (124/315 [39 %] children versus 154/288 [53 %] children, *P <* 0.05), FCT (1.1 ± 0.3 days [95 % *CI* 1–1.1 *n* = 234] versus 1.2 ± 0.7 days [95 % *CI* 1.1–1.3, *n* = 222]; *P* = 0.002), and PCT (1.4 ± 0.6 days [95 % *CI* 1.4–1.5] versus 1.6 ± 0.6 days [95 % *CI* 1.5–1.7]; *P <* 0.05) were significantly lower in children treated with AA compared with those treated with AL. The PRR 1 day after treatment began was significantly higher in children treated with AA compared with those treated with AL (1.9 × 10^3^ [range 3.2 × 10^−1^–1.1 × 10^6^] versus 5.6 × 10^2^ [range 9.5 × 10^−1^–2.1 × 10^6^]; *P <* 0.05). However, the PCR-corrected ACPR on day 28 (294/310 [95 %] versus 265/279 [95 %] for AA and AL, respectively; *P* >0.05) and the AnRT (10.4 days [95 % *CI* 9–11.8] versus 11 days [95 % *CI* 9.4–12.6] for AA and AL, respectively; *P* >0.05) were similar for the two treatment groups.

### Kinetics of the disposition of parasitaemia

In children aged 2 years and under, the kinetics of the disposition of parasitaemia were evaluated in 20 children with the following demographic and clinical characteristics: mean age = 1.6 years (range 0.6–2), male/female ratio 13:7, mean duration of illness = 2.3 days (range 1–4), mean body temperature = 38.1 °C (range 36.6–39.9 °C, no. with > 37.4 °C = 12), mean HCT = 28 % (range 20–38, no. with < 30 % = 12), geometric mean asexual parasitaemia of 48,580 μL^−1^ (range 3625–288,461; no. with > 100,000 μL^−1^ = 5; no. with > 250,000 μL^−1^ = 1), mean FCT = 1.1 days (range 1–2), mean PCT = 1.2 days (range 1–3), and mean AnRT 13.5 days (range 1–28).

**Fig. 3:**
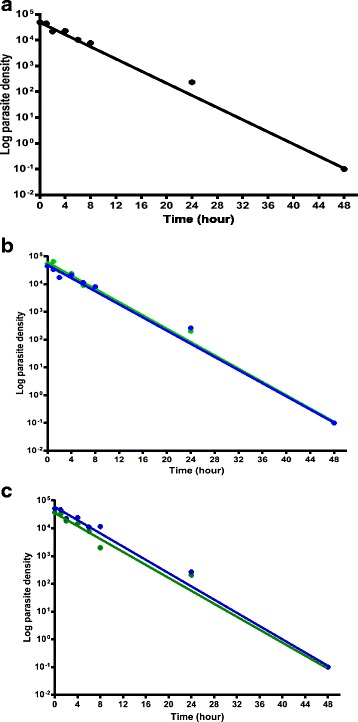
Semilog plots of parasitaemia versus time (**a**) in all, (**b**) in AA (*blue*) and AL (*green*)-treated children and (**c**) in ≤ 1-year olds (*blue*) and > 1-year olds (*green*). [AA, artesunate-amodiaquine; AL, artemether-lumefantrine]

Overall, a monoexponential decline of parasitaemia was observed, with an estimated mean elimination half-time (t_½el_) of 1 h (95 % *CI* 0.9–1.1) (see Fig. 3a). The estimated mean t_½el_ values were similar for AA (1 h, 95 % *CI* 0.9–1.2)- and AL (0.9 h, 95 % *CI* 0.9–1.7)- treated children (*P* >0.05) (see Fig. 3b). In children aged 1 year old and under (*n* = 3) and those older than 1 year (*n* = 17), the estimated mean elimination half-times were 0.9 h (95 % *CI* 0.2–1.5) and 1 h (95 % *CI* 0.9–1.6), respectively; they were similar (*P* >0.05) (see Fig. 3c). Estimated mean elimination half-times were also similar in children with enrolment parasitaemia ≥ 100 000 μL^−1^ and those with < 100 000 μL^−1^ (1 h [95 % *CI* 0.6–1.4, *n* = 5] versus 1 h [95 % *CI* 0.9–1.1, *n* = 15]; *P* >0.05, respectively) and in anaemic and non-anaemic children at presentation (1 h [95 % *CI* 0.8–1.1, *n* = 11] versus 1 h [95 % *CI* 0.7–1.2, *n* = 9]; *P* >0.05, respectively).

### Relationship between elimination half-time of parasitaemia and PCT

The relationship between the elimination half-time of parasitaemia and PCT in the same patients was evaluated in 20 children aged 2 years old and under. The mean elimination half-time of parasitaemia was 1 h (95 % *CI* 0.9–1.1) and PCT was 1.1 day (95 % *CI* 1–1.2). A significantly positive correlation was observed between elimination half-time of parasitaemia and PCT in the same patients (*r* = 0.64, *P* <0.05), however, no correlation was observed between elimination half-time of parasitaemia and PRRs 1 or 2 days after treatment began (*r* = 0.42, *P* >0.05 and *r* = 0.21, *P* >0.05, respectively).

### Adverse events

Adverse events were carefully monitored at the Ibadan study site. Thirteen of the 61 children aged 2 years and under (8/26 on AA and 5/35 on AL) reported at least one adverse event within 1 week of starting treatment. There was no significant difference in the proportions of children reporting adverse events between the two treatment groups (*P* >0.05). Overall, fever (*n* = 7) and cough (*n* = 7) were the most frequently reported adverse events in children treated with AA and AL. Abdominal pain (*n* = 1) and weakness (*n* = 1) were the least reported adverse events; they were reported only in the group treated with AA. Almost all the reported adverse events occurred in children aged older than 1 year.

Fifty-five out of 241 children older than two but younger than 5 years (40/128 on AA and 15/113 on AL) reported at least one adverse event within 1 week of starting treatment. There was a significant difference in the proportions of children reporting adverse events between the two treatment groups (*P* <0.05). Overall, there was no significant difference in the reported adverse events in those aged 2 years and under and those older than two but younger than 5 years old (*P* >0.05).

Overall, fever (*n* = 29) and cough (*n* = 23) were the most frequently reported adverse events in children treated with AA and AL. Weakness (*n* = 4) was the least reported adverse event.

### Recovery from anaemia

In children aged 2 years and under, anaemia was present at enrolment in 127 children, and was mild, moderate or severe in 112 (88 %), 14 (11 %) and 1 (1 %) children, respectively. All but 13 children with anaemia at presentation recovered from their anaemia. Of the 13 children who did not recover from their anaemia, 10 and three children had mild and moderate anaemia, respectively. In the 114 children who recovered from anaemia, the mean AnRT was 12.1 days (95 % *CI* 10.6–13.6) and was similar for the two treatment groups (12.2 days [95 % *CI* 9.9–14.4, *n* = 60] versus 12.1 days [95 % *CI* 10.0–14.2, *n* = 54] in AA- and AL-treated children, respectively [*P* >0.05]). The AnRT was also similar in children aged 1 year and under and those aged 1 year and over (13.1 days [95 % *CI* 9.7–16.5, *n* = 23] versus 11.9 days [95 % *CI* 10.1–13.6, *n* = 91]; *P* >0.05). Overall, the mean AnRT was significantly longer in children with high enrolment parasitaemia (≥100,000 μL^−1^) compared with those with low enrolment parasitaemia (<100,000 μL^−1^) (19.1 days [95 % *CI* 10.5–27.7, *n* = 7] versus 11.7 days [95 % *CI* 10.1–13.2, *n* = 107]; *P* = 0.02). A significantly positive correlation was observed between AnRT and enrolment parasitaemia (*r* = 0.24, *P* <0.05, *n* = 114), but there was no correlation between AnRT and age (*r* = 0.15, *P* <0.05, *n* = 114) or HCT at presentation (*r* = 0.11, *P* >0.05, *n* = 114) in the same patients.

### Late-appearing anaemia following treatment

Eleven children aged 2 years and under (4.4 %) had LAA (see Table 7). The mean elapsed time from the start of treatment to appearance of late anaemia was 24.2 days (range 21–28 days). None of the 11 children reported a symptom at the time of LAA, but one child had a body temperature of 39.5 °C at the time of appearance of late anaemia. Three out of five children treated with AA had > 11 mg/kg total dose of artesunate. Five children did not recover from their LAA; these children were followed up for 28 days.

**Table 7:** Features of ≤ 2 years old children who developed anaemia after day 14

Patient (gender, age)	Year of enrolment	Parasitaemia (μL^−1^)	Enrolment HCT (%)	Antimalarial treatment	PCT (day)	Nadir HCT (%) [day]	Time of LAA (day)	LAnRT	Parasite reduction ratio (PRR)		Total of mg/kg dose of AA		Total of mg/kg dose of AL	
									Day 1	Day 2	Artesunate	Amodiaquine	Artemether	Lumefantrine
124 (M, 2y)	2010	12,648	25	AL	2	28 [28]	28	NA^c^	1.2 × 10^1^	1.3 × 10^4^	NA	NA	10.9	65.5
15 (M, 1.2y)	2008	72,815	27	AL	1	28 [28]	21	14	7.3 × 10^4^	7.3 × 10^4^	NA	NA	17.4	102
46 (M, 0.8y)	2015	16,979	27	AA	1	29 [21]	21	7	1.7 × 10^4^	1.7 × 10^4^	15	40.5	NA	NA
58 (M, 2y)	2010	19,647	28	AL	4	28 [28]	28	NA^c^	7.6 × 10^0^	2.5 × 10^2^	NA	NA	8.6	51.4
15 (M, 1y)	2014	29,714	30	AL	1	29 [28]	28	7	3.0 × 10^4^	3.0 × 10^4^	NA	NA	8.6	51.4
100 (M, 1.3y)	2010	118,344	30	AL	2	29 [21]	21	7	3.5 × 10^1^	1.2 × 10^5^	NA	NA	13.3	80
64 (F, 1.9y)^a^	2010	76,000	30	AA	2	29 [28]	28	NA^c^	5.5 × 10^1^	7.6 × 10^4^	14.3	38.6	NA	NA
8 (F, 1y)	2010	25,242	30	AA	2	28 [28]	28	NA^c^	2.4 × 10^2^	2.5 × 10^4^	10.7	28.9	NA	NA
40 (F, 2y)	2010	1,652	30	AA	1	28 [21]	21	7	1.7 × 10^3^	1.7 × 10^3^	9.4	25.3	NA	NA
911 (M, 1.9y)	2011	2,924	30	AA	1	28 [21]	21	14	2.9 × 10^3^	2.9 × 10^3^	12.5	38.3	NA	NA
43 (M, 1.3y)	2010	1,301	31	AL	1	27 [28]	21	NA^c^	1.3 × 10^3^	1.3 × 10^3^	NA	NA	13.3	80
Mean														
Age (1.5y)		15,569^b^	28.9		1.6	28.3	24.2	9.3	6.6 × 10^2^	1.0 × 10^4^	12.4	34.3	12	71.7

*HCT* haematocrit, *PCT* parasite clearance time, *M* male, *F* female, *AA* artesunate-amodiaquine, *AL* artemether-lumefantrine, *LAA* late-appearing anaemia, *LAnRT* time to recovery from late-appearing anaemia, *NA* not applicable

^a^child with fever of 39.5 °C on day 28

^b^Geometric mean parasitaemia

^c^follow up was limited to 28 days

## Discussion

In this study, both AA and AL were proved to be effective treatments for uncomplicated falciparum malaria, as evidenced by a PCR-corrected parasitological efficacy of over 96 %. Both treatments were similar in their therapeutic efficacy. In children older than 2 years of age, both treatments rapidly reduce parasitaemia and fever compared with children aged 2 years and below. These findings suggest that therapeutic responses to both ACTs were not uniform across the age range of below 5 years and may be influenced by age. Children older than 2 years may possess some relative degree of immunity compared with very young children.

Among the children older than 2 years, AA was superior in clearing parasitaemia and fever. This would suggest that the superior efficacy of AA over AL in clearing parasitaemia and fever in children younger than 5 years of age in this endemic area [17, 18, 20] can, to a large extent, be attributable to the effects of AA on parasitaemia and fever clearance in children older than 2 years. Children older than 2 years form the bulk of the children under 5 years of age that are usually included in therapeutic efficacy studies in endemic areas. The small number of patients (3/250 children ≤ 2 years and 7/603 children >2– < 5) who exhibited parasite positivity on day 3 indicates that there is no evidence for slow clearance of parasitaemia in very young and older children in this endemic area. The low parasite positivity on day 3 is in contradiction to the situation in the Greater Mekong subregion, where parasite positivity on day 3 is high in children treated with artemisinin [3, 7].

The monoexponential declines of parasitaemia indicated that the disposition of parasitaemia following treatment with both ACTs is a first-order process. The parasitaemia elimination half-time of 1 h provides a baseline for which future changes in parasite population in vivo and in vitro susceptibility profile in very young children from this endemic area may be measured or compared. It is noteworthy that the elimination half-time of parasitaemia of 1 h is considerably lower than the 5.5 h reported in areas in the Greater Mekong subregion where resistance to artemisinin has emerged [9].

Reported adverse events following treatment were few, and were similar for the two ACTs. The relative lack of reported adverse events in very young children can be attributed to their inability to name them. In children older than 2 years, the reported adverse events were significantly more frequent in those children treated with AA compared with those treated with AL. This finding is similar to a recent study conducted in older patients, where reported adverse events were more frequent in those treated with AA compared with AL [29].

Virtually all children who were anaemic at presentation recovered from their anaemia within 2 weeks of starting treatment, a time similar to that previously reported in a cohort study of children aged younger than 5 years [20]. An interesting finding was the relatively asymptomatic nature of LAA, which occurred in 4 % of very young children. It has been reported that symptomatic delayed haemolytic anaemia can occur following intravenous artesunate treatment of severe malaria in immunological naïve patients [30–32] and in African children [33], and is attributable to a delayed destruction of once-parasitized red blood cells from which the parasites have been removed by ‘pitting’ during passage through the spleen [34]. The relatively asymptomatic nature of and the paucity of signs during LAA may be responsible for the failure to recognize LAA as a ‘saving’ effect of ACTs due to rapid clearance of parasitaemia and conservation of HCT. The children who did not recover from their LAA were followed up for 28 days. It is now necessary to follow up children treated with ACTs for 42 days in order to detect and ensure that children with LAA recover from it. Thus, LAA may have practical implications in endemic areas where ACTs have become first-line treatments for uncomplicated malaria and where anaemia is a significant contributor to morbidity.

There limitations of the present study, in particular with late-appearing anaemia. These include non-characterization of the haemolytic nature of the late-appearing anaemia and non-evaluation of the contributions of background causes of anaemia in this endemic area to the late-appearing anaemia.

## Conclusion

This study found that AA and AL are both efficacious treatments for uncomplicated falciparum malaria in Nigerian children aged 2 years but therapeutic responses to the two ACTs are not uniform across the age band 6–59 months. Both treatments may cause a relatively asymptomatic LAA with uneventful recovery in a small proportion of very young children.

## Abbreviations

AA, artesunate-amodiaquine; ACPR, adequate clinical and parasitological response; ACT, artemisinin-based combination therapy; AL, artemether-lumefantrine; ANOVA, analysis of variance; AnRT, anaemia recovery time; FCT, fever clearance time; HCT, haematocrit; LAA, late-appearing anaemia; LPF, late parasitological failure; PCR, polymerase chain reaction; PCT, parasite clearance time; PPR, parasite reduction ratio; PRR_D1_, parasite reduction ratio 1 day after treatment began; PRR_D2_, parasite reduction ratio 2 days after treatment began; SEM, standard error of mean; t_½_, half-time

## Additional file

Additional file 1: Multilingual abstracts in five official working languages of the United Nations. (PDF 403 kb)
