# Supplementary material for: Therapeutic efficacy and effects of artesunate-amodiaquine and artemether-lumefantrine on malaria-associated anaemia in Nigerian children aged two years and under
Source: Infect Dis Poverty. 2016 Jul 6;5:70. doi: 10.1186/s40249-016-0165-2 (PMC4933999; doi:10.1186/s40249-016-0165-2)
Supplement: Additional file 1: — Multilingual abstracts in five official working languages of the United Nations. (PDF 403 kb) [file 40249_2016_165_MOESM1_ESM.pdf]

Translation of the abstract into the five official working languages of the United Nations

النجاعة العلاجية وآثار الأرتيسونات-أمودياكين وأرتيميثير-اللوميفانترين على فقر الدم المصاحب للملاريا عند الأطفال النيجيريين الذين تتراوح أعمارهم بين عامين و الفئات العمرية تحت ذلك.

lsie O. Adewoye, Bayo Fatunmbi, m, EAkintunde Sowunmi, Kazeem Akano, Adejumoke I. Ayede, Godwin Ntado and Temitope Aderoyeje

الملخص

معلومات أساسية: تعتبر العلاجات المبنية على الجمع بين أحد العقاقير والأرتيميسينين الخطوة الأولى المنصوح باستعمالها في علاج الملاريا المنجلية غير معقدة، ولكن فعالية وآثار هذه العقاقير على فقر الدم غير معقدة المصاحبة للملاريا عند الأطفال الذين تتراوح أعمارهم بين عامين والفئات العمرية الأقل من ذلك لم تتم التحقق منها بالشكل الكافي.

الوسائل: تم تقييم فعالية وآثار العقاقير المستخرجة من الطفيليات على فقر الدم المصاحب الملاريا عند 250 طفلا مصابا بالملاريا الذين تتراوح أعمارهم بين عامين والفئات العمرية تحت ذلك، وكذلك تقييم فعالية العقار عند 603 أطفال مصابا بالملاريا التي تتراوح أعمارهم بين 2 و5 سنوات بعد العلاج بالأرتيسونات-أمودياكين (AA) أو أرتيميثير-اللوميفانترين (AL). تم تقييم حركية التخلص من الطفيليات في الدم بعد العلاج باستخدام: non-compartment model. تم تشخيص فقر الدم المتأخر الظهور (LAA)باستخدام المعايير التالية: تنقية الدم من الطفيليات والحمى والأعراض الأخرى التي تحدث خلال سبعة أيام من بدء العلاج، والاستجابة السريرية والطفيليات الكافية بين اليوم 28 إلى اليوم 42،(HTC)الهيماتوكريت أكبر أو يساوي 30٪ خلال أسبوع أو أسبوعين ، انخفاض في الهيماتوكريت إلى أقل من 30٪ التي تحدث خلال 3-6 أسابيع، وغياب مرض مرافق بين أسبوع إلى 6 أسابيع، وغياب الطفيل اللاجنسي الذي تم الكشف عنه باستخدام كل من المجهر و وتفاعل البوليميريز المتسلسل (PCR) بين أسبوع و إلى 6 أسابيع.

النتائج: إجمالا، عند الأطفال الذين تتراوح أعمارهم بين عامين والفئات العمرية تحت ذلك، فعالية الطفيليات المعدلة باستعمال تفاعل البوليميرز المتسلسل إجمالا هو 97.2٪ (95٪ CI 8،92-6،101)، وهي نفس النسبة في كلتا العلاجين. عند الأطفال التي تتراوح أعمارهم بين سنتين فما فوق، كانت فعالية الطفيليات أيضا مماثلة لكلا العلاجين، ولكن انتشار الطفيليات بعد يوم واحد من العلاج كان أسرع، وكانت الحمى وفترة تنقية الدم من الطفيليات أسرع بكثير عند الأطفال المعالجين بالأرتيسونات-أمودياكين (AA) مقارنة مع الأطفال المعالجين بأرتيميثير-اللوميفانترين (AL). انخفاض الحمل الطفيلي بشكل أسي أحادي (monoexponential) وبلغت فترة عمر النصف (time-half) مدة ساعة واحدة. فترة عمر النصف(time-half) كانت متساوية في كلتا العلاجين. عند الأطفال الذين تتراوح أعمارهم بين عامين والفئات العمرية تحت ذلك و الذين يعانون من فقر الدم في وقت عرض الدراسة،المتوسط الحسابي لفترة التعافي من فقر الدم هو 12.1 يوما (95٪ CI 6،10-6،13، ن = 127)، و وهي نفس المدة في كلتا العلاجين. فقر الدم المتأخر الظهور(LAA) و الذي يتجلى نسبيا بدون أعراض عند 11 طفلا (4.4٪) الذين تتراوح أعمارهم بين عامين والفئات العمرية تحت ذلك، وفي هذه الحالة كان التعافي سلسا دون شيئ يذكر .

استنتاج: أظهرت هذه الدراسة أن العلاجين بالأرتيسونات-أمودياكين (AA) أو أرتيميثير-اللوميفانترين (AL) فعالين لمكافحة الملاريا المنجلية غير المصحوبة بمضاعفات عند الأطفال النيجيريين الذين تتراوح أعمارهم بين عامين والفئات العمرية تحت ذلك، وأن العلاج بالأرتيسونات-أمودياكين (AA) ينقي الدم من الطفيليات ويعالج الحمى أسرع بكثيرمن العلاج بأرتيميثير-اللوميفانترين(AL) عند الأطفال التي تتراوح أعمارهم بين سنتين فما فوق. قد يسبب العلاجين على حد سواء فقر الدم المتأخر الظهور(LAA) والذي يتجلى نسبيا بدون أعراض مقرون مع مرحلة تعافي عادية عند نسبة صغيرة من الأطفال الذين تتراوح أعمارهم بين عامين والفئات العمرية تحت ذلك.

Translated from English version into Arabic by Aziz Hamdoun, through

TRANSLATORS
WITHOUT BORDERS

青蒿琥酯-阿莫地喹和蒿甲醚-苯芴醇对尼日利亚 2 岁及以下儿童疟疾性贫血的疗效和作用研究

Akintunde Sowunmi, Kazeem Akano, Adejumoke I. Ayede, Godwin Ntadom, Elsie O. Adewoye,Bayo Fatunmbi, and Temitope Aderoyeje

摘要

引言：以青蒿素为基础的联合疗法是无并发症恶性疟的首选治疗方法，但目前几乎未见对 2 岁及以下儿童无并发症疟疾性贫血的疗效和作用的评价研究。

方法：本研究评价了青蒿琥酯-阿莫地喹（AA）或蒿甲醚-苯芴醇（AL）对 250 例 2 岁及以下疟疾患儿的寄生虫学疗效和作用，同时评估了上述两种联合疗法对 603 例 2～5 岁患儿的疗效。采用非房室模型分析治疗时虫血症的处置动力学。通过如下标准诊断继发性贫血：虫血症清除，治疗后 7 d 出现发热或其他症状，28-42 d 出现适当的临床和寄生虫学反应，1～2 周红细胞压积（HCT）≥30%，3～6 周 HCT 降至 30%以下，1～6 周无伴随疾病发生，1～6 周通过显微镜和 PCR 未检测到无性期虫血症。

结果：总的来说，采用两种疗法进行治疗的 2 岁及以下儿童 PCR 校正的寄生虫学治愈率为 97.2%(95% *CI*：92.8–101.6)，疗效相近。大于 2 岁的儿童虽然寄生虫学治愈率亦相近，但与 AL 相比，使用 AA 治疗后 1 d 的患病率更高、发热更早、清除所需时间更短。虫血症的下降与预估的 1 h 消除半衰期呈单指数关系。两种疗法的消除半衰期相近。在已发贫血的 2 岁及以下儿童中，贫血平均恢复期为 12.1 d(95% *CI*：10.6–13.6, *n* = 127)，两种治疗方法无显著性差异。2 岁及以下儿童中有 11 名（4.4%）出现无症状的继发性贫血，但恢复平稳。

结论：本研究显示 AA 和 AL 对尼日利亚 2 岁及以下儿童的无并发症恶性疟均有效，2 岁以上儿童中 AA 比 AL 可更快消除虫血症和发热症状。在 2 岁及以下儿童中两种疗法均会引起小比例的无症状且恢复平稳的继发性贫血。

Translated from English version into Chinese by Song Peng, edited by Yang Pin, through

Efficacité thérapeutique et effets des combinaisons artésunate-amodiaquine et artéméther-luméfantrine sur l'anémie palustre chez des enfants nigérians âgés de deux ans et moins

Akintunde Sowunmi, Kazeem Akano, Adejumoke I. Ayede, Godwin Ntadom, Elsie O. Adewoye, Bayo Fatunmbi et Temitope Aderoyeje

Résumé

Contexte : Bien que les thérapies combinées à base d'artémisinine constituent la première ligne de traitement recommandée contre le paludisme à *P. falciparum*, leur efficacité et leurs effets sur l'anémie palustre non compliquée a été peu évaluée chez les nourrissons jusqu'à l'âge de deux ans.

Méthodes : L'efficacité parasitologique et les effets sur l'anémie palustre du traitement combiné artésunate-amodiaquine (AA) ou artéméther-luméfantrine (AL) ont été évalués chez 250 enfants âgés de deux ans ou moins atteints de paludisme et son efficacité chez 603 enfants âgés de 2 à 5 ans. La cinétique d'élimination de la parasitémie après le traitement a été évaluée à l'aide d'un modèle non compartimenté. L'anémie d'apparition tardive (AT) a été diagnostiquée selon les critères suivants : élimination de la parasitémie, de la fièvre et des autres symptômes dans les sept jours suivant le début du traitement, réponse clinique et parasitologique adéquate à J28-J42, hématocrite (HCT) ≥ 30 % à une et/ou deux semaines, baisse de l'hématocrite à < 30 % entre 3 et 6 semaines, absente de

comorbidité entre 1 et 6 semaines et absence de parasitémie asexuée détectée à la fois par microscopie et par réaction en chaîne à la polymérase (RCP) entre 1 et 6 semaines.

Résultats : Dans l'ensemble, chez les nourrissons de deux ans ou moins, l'efficacité parasitologique corrigée par la RCP était de 97,2 % (*IC* à 95 % de 92,8 à 101,6) et elle était similaire avec les deux traitements. Chez les enfants de plus de deux ans, elle était similaire aussi mais la prévalence du parasite un jour après le début du traitement était nettement supérieure et les temps d'élimination de la fièvre et de la parasitémie significativement plus courts chez les enfants du groupe AA par rapport au groupe AL. La baisse de la parasitémie était monoexponentielle, avec une demi-vie d'élimination estimée d'une heure. Les demi-vies d'élimination étaient similaires avec les deux traitements. Chez les enfants âgés de deux ans et moins anémiés à la présentation, le délai moyen de récupération de l'anémie était de 12,1 jours (*IC* à 95 % de 10,6 à 13,6, *n* = 127) et comparable avec les deux traitements. Une AT relativement asymptomatique s'est produite chez 11 enfants (4,4 %) âgés de deux ans ou moins et s'est résolue sans complications.

Conclusion : Cette étude a montré que les modalités AA et AL constituent des traitements efficaces contre le paludisme à *P. falciparum* non compliqué chez des enfants nigérians âgés de deux ans et moins et que le traitement AA élimine la parasitémie et la fièvre significativement plus vite que la modalité AL chez les enfants âgés de plus de 2 ans. Les deux traitements peuvent causer une anémie d'apparition tardive, relativement asymptomatique et se résolvant sans complications, chez un petit pourcentage d'enfants âgés de deux ans ou plus.

Translated from English version into French by Suzanne Assenat, through

TRANSLATORS
WITHOUT BORDERS

Терапевтическая эффективность и воздействие артесунат-амодиахина и артеметер-люмефантрина на вызванную малярией анемию у нигерийских детей в возрасте двух лет и младше

Акинтунде Совунми, Казим Акано, Адеюмоке И. Айеде, Годвин Нтадом, Элси О. Адевойе, Байо Фатунмби и Темитопе Адеройехе

Резюме

Общие сведения: Артемизинин-комбинированная терапия (АКТ) рекомендуется в качестве наилучшего метода лечения неосложненной молниеносной малярии, но дополнительная информация об эффективности ее применения и воздействия на неосложненную молниеносную малярию у детей в возрасте двух лет и младше практически отсутствует.

Методы: Исследование паразитологической эффективности и влияния на анемию, вызванную малярией, было проведено у 250 детей, больных малярией, в возрасте двух лет и младше. Также была проведена оценка эффективности лечения комбинацией артесуната и амодиахина (АА) либо артеметера и люмефантрина (АЛ) 603 детей, страдающих малярией, в возрасте от двух до пяти лет. Кинетика падения уровня паразитемии после лечения оценивалась без использования камерной модели. Анемия с поздним началом была диагностирована с использованием следующих критериев: исчезновение паразитемии, лихорадки, а также других симптомов в пределах семидневного периода после начала лечения, удовлетворяющий требованиям клинический и паразитологический ответ на 28-42 день, НСТ крови ≥ 30% в первую и/или вторую неделю, снижение НСТ крови до < 30% происходит на 3–6 неделю, отсутствие сопутствующих заболеваний в 1-6

неделю, отсутствие паразитемии, подтвержденное как микроскопической диагностикой, так и полимеразной цепной реакцией (ПЦР) в 1- 6 неделю.
Результаты: В целом, у детей в возрасте двух лет и младше паразитологическая эффективность (терапии) с учетом данных ПЦР-анализа составила 97.2% (95% ДИ 92.8–101.6) и была приблизительно одинакова в обоих случаях лечения. У детей в возрасте двух лет и младше паразитологическая эффективность также была приблизительно одинакова для обоих методов лечения, но паразитарная нагрузка через один день после начала лечения была значительно выше, а повышенная температура и паразитемия проходили значительно быстрее у детей, принимающих препарат артесунат-амодиахин (АА) по сравнению с детьми, принимающими препарат артеметер-люмефантрин (АЛ). При паразитемии концентрация (препарата) снижалась моноэкспоненциально, расчетный период полувыведения (препарата из организма) составлял 1 час. Расчетный период полувыведения (препарата из организма) в обоих случаях лечения был одинаковым. У детей в возрасте двух лет и младше, поступивших с анемией (у которых при поступлении отмечалась анемия), средний срок выздоровления от анемии составлял 12,1 дней (95% ДИ 10.6–13.6, n = 127) и был одинаков для обоих методов лечения. Сравнительно бессимптомная анемия с поздним началом, не сопровождающаяся выраженной клинической симптоматикой, отмечалась у 11 детей в возрасте 2 лет и младше и прошла/разрешилась без осложнений.
Заключение: Данное исследование показало, что артесунат-амодиахин (АА) и артеметер-люмефантрин (АЛ) являются эффективными для лечения неосложненной молниеносной малярии у нигерийских детей в возрасте двух лет и младше, а также, что артесунат-амодиахин (АА) устраняет паразитемию и повышенную температуру значительно быстрее, чем артеметер-люмефантрин (АЛ) у детей в возрасте двух лет и младше. Оба препарата могут вызывать (приводить к развитию) анемию с поздним началом, которая не сопровождается выраженной клинической симптоматикой и проходит (разрешается) без осложнений.

Translated from English version into Russian by Tatsiana Mankevich, through

TRANSLATORS
WITHOUT BORDERS

Eficacia terapéutica y efectos del artesunato-amodiaquina y del artemeter-lumefantrina para el tratamiento de la anemia palúdica en niños nigerianos de hasta dos años de edad

Akintunde Sowunmi, Kazeem Akano, Adejumoke I. Ayede, Godwin Ntadom, Elsie O. Adewoye, Bayo Fatunmbi, and Temitope Aderoyeje

Resumen
Antecedentes: Las terapias combinadas con artemisina se recomiendan como tratamiento de elección del paludismo por *Plasmodium falciparum*, pero apenas se ha evaluado su eficacia y sus efectos en la anemia palúdica sin complicaciones en niños de hasta dos años de edad.
Métodos: La eficacia parasitológica y los efectos del tratamiento de la anemia palúdica con artesunato-amodiaquina (AA) o artemeter-lumefantrina (AL) se evaluaron en 250 niños palúdicos de hasta dos años de edad, y la eficacia se evaluó en 603 niños palúdicos de 2 a 5 años de edad. La cinética de eliminación de la parasitemia tras el tratamiento se evaluó mediante un modelo no compartimental. La anemia de aparición tardía (LAA, por sus siglas en inglés) se

diagnosticó según los siguientes criterios: eliminación de la parasitemia, fiebre y otros síntomas que se den durante los siete días después de empezar el tratamiento, una respuesta clínica y parasitológica apropiada entre los días 28 y 42, valores de hematocrito (HCT) ≥ 30 % en la primera o segunda semana, una caída de los valores de HCT a menos del 30 % entre la tercera y sexta semana, la ausencia de enfermedades concomitantes entre la primera y sexta semana, y la ausencia de parasitemia asexual detectada mediante microscopía y reacción en cadena de la polimerasa (PCR, por sus siglas en inglés) entre la primera y sexta semana.

Resultados: A grandes rasgos, en los niños de hasta dos años de edad, la eficacia parasitológica corregida con datos de la PCR fue del 97,2 % (95 % *CI* 92,8–101,6), lo que resultó similar para ambos tratamientos. En los niños de más de dos años de edad, la eficacia parasitológica fue también similar en ambos tratamientos, pero la prevalencia parasítica un día después de empezar el tratamiento fue significativamente más elevada, y la eliminación de la fiebre y los parásitos resultaron significativamente más rápidos en los niños tratados con AA que en los niños tratados con AL. La reducción de la parasitemia fue monoexponencial con una semivida de eliminación de una hora. Las semividas de eliminación fueron similares para ambos tratamientos. En niños de hasta dos años de edad que presentaban anemia en el momento de iniciar el tratamiento, el tiempo de recuperación promedio de la anemia fue de 12,1 días (95 % *CI* 10,6–13,6, *n* = 127), lo que fue similar para ambos tratamientos. Once niños (4,4 %) de edades de hasta dos años presentaron LAA relativamente asintomática, de la que se recuperaron sin ningún problema.

Conclusión: Con este estudio se muestra que el AA y el AL constituyen tratamientos eficaces del paludismo por *Plasmodium falciparum* sin complicaciones en niños nigerianos de hasta dos años de edad, y que el AA elimina la parasitemia y la fiebre con mucha más rápidez que el AL en los niños de más de dos años. Ambos tratamientos pueden causar una LAA relativamente asintomática con una recuperación sin problemas en una pequeña proporción de los niños de hasta dos años de edad.

Translated from English version into Spanish by Mar Jiménez Quesada, through

TRANSLATORS
WITHOUT BORDERS
